# Computational simulation model of transcatheter edge-to-edge mitral valve repair: a proof-of-concept study

**DOI:** 10.1093/ehjci/jeae137

**Published:** 2024-05-27

**Authors:** David Messika-Zeitoun, Jamal Mousavi, Mohammad Pourmoazen, Florian Cotte, Julien Dreyfus, Mohammed Nejjari, David Attias, Martin Kloeckner, Said Ghostine, Romain Pierrard, Armand Eker, Franck Levy, Yvan Le Dolley, Remi Houel, Rudy R Unni, Ian G Burwash, Adam Dryden, Mark Hynes, Donna Nicholson, Marino Labinaz, Vincent Chan, Jean-Noel Albertini, Thierry Mesana

**Affiliations:** Division of Cardiology, University of Ottawa Heart Institute, 40 Ruskin Street, Ottawa, Ontario, Canada K1Y 4W7; Predisurge, Saint-Etienne, France; Predisurge, Saint-Etienne, France; Predisurge, Saint-Etienne, France; Department of Cardiology, Centre Cardiologique du Nord, Saint-Denis, France; Hemodynamic Department, Centre Cardiologique du Nord, Saint-Denis, France; Department of Cardiology, Centre Cardiologique du Nord, Saint-Denis, France; Groupe Hospitalier Paris Saint Joseph, Hôpital Marie Lannelongue, Le Plessis Robinson, France; Groupe Hospitalier Paris Saint Joseph, Hôpital Marie Lannelongue, Le Plessis Robinson, France; Cardiology Department, Saint Etienne University Hospital, Saint-Etienne, France; Centre Cardiothoracique de Monaco, Monaco; Centre Cardiothoracique de Monaco, Monaco; Percutaneous Therapy Valve Unit, Hôpital Saint Joseph, Marseille, France; Percutaneous Therapy Valve Unit, Hôpital Saint Joseph, Marseille, France; Division of Cardiology, University of Ottawa Heart Institute, 40 Ruskin Street, Ottawa, Ontario, Canada K1Y 4W7; Division of Cardiology, University of Ottawa Heart Institute, 40 Ruskin Street, Ottawa, Ontario, Canada K1Y 4W7; Division of Anesthesiology, University of Ottawa Heart Institute, Ottawa, Canada; Division of Anesthesiology, University of Ottawa Heart Institute, Ottawa, Canada; Division of Anesthesiology, University of Ottawa Heart Institute, Ottawa, Canada; Division of Cardiology, University of Ottawa Heart Institute, 40 Ruskin Street, Ottawa, Ontario, Canada K1Y 4W7; Division of Cardiac Surgery, University of Ottawa Heart Institute, Ottawa, Canada; Predisurge, Saint-Etienne, France; Centre Cardiothoracique de Monaco, Monaco; Division of Cardiac Surgery, University of Ottawa Heart Institute, Ottawa, Canada

**Keywords:** mitral regurgitation, transcatheter valve intervention, computational simulation

## Abstract

**Aims:**

As transcatheter mitral valve (MV) interventions are expanding and more device types and sizes become available, a tool supporting operators in pre-procedural planning and the clinical decision-making process is highly desirable. We sought to develop a finite element computational simulation model to predict the results of transcatheter edge-to-edge repair (TEER) interventions.

**Methods and results:**

We prospectively enrolled patients with secondary mitral regurgitation (MR) referred for a clinically indicated TEER. The 3D trans-oesophageal echocardiograms performed at the beginning of the procedure were used to perform the simulation. On the 3D dynamic model of the MV that was first obtained, we simulated the clip implantation using the same clip type, size, number, and implantation location that was used during the intervention. The 3D model of the MV obtained after the simulation of the clip implantation was compared with the clinical results obtained at the end of the intervention. We analysed the degree and location of residual MR and the shape and area of the diastolic MV area. We performed computational simulation on five patients. Overall, the simulated models predicted well the degree and location of the residual regurgitant orifice(s) but tended to underestimate the diastolic mitral orifice area.

**Conclusion:**

In this proof-of-concept study, we present preliminary results on our algorithm simulating clip implantation in five patients with functional MR. We show promising results regarding the feasibility and accuracy in terms of predicting residual MR and the need to improve the estimation of the diastolic MV area.

## Graphical Abstract

**Graphical Abstract:**
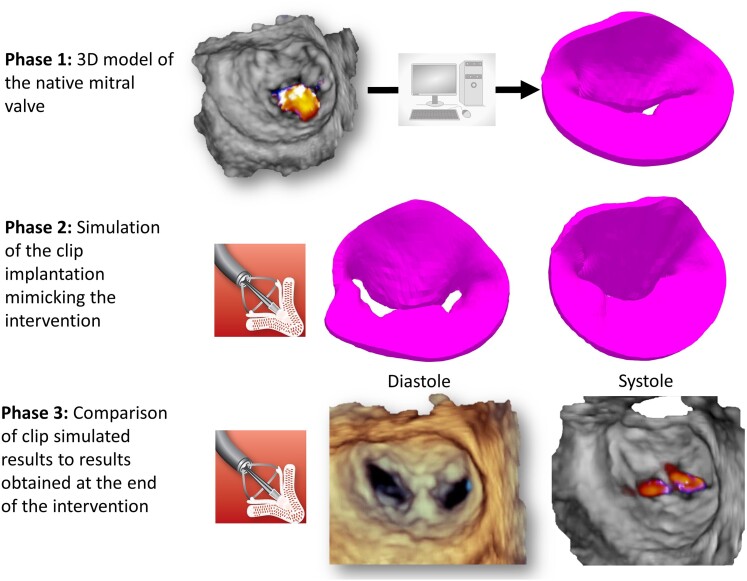
Schematic view of the study design and the simulation’s phases.

## Introduction

Transcatheter edge-to-edge repair (TEER) is currently recommended in selected patients with mitral regurgitation (MR) considered at high surgical risk.^1,2^ Although the level of evidence for improved survival is less robust for secondary MR than for primary MR due to discordant results in the two available randomized controlled trials, evidence of benefit is accumulating, and the number of TEER interventions is rapidly increasing.^3–6^ In contrast to aortic valve stenosis and transcatheter aortic valve implantation, the mitral valve (MV) is a more complex structure with multiple underlying potential mechanisms and aetiologies responsible for the regurgitation, necessitating personalized interventions. Improvements in technology and the availability of more device types and sizes will increase the therapeutic options, and planning for mitral interventions will become increasingly complex.^7^ A tool capable of predicting the results of transcatheter MV interventions and helping guide the selection of device type, size, and positioning, i.e. the pre-procedural planning and clinical decision-making, is highly desirable.^8,9^ Importantly, TEER is currently performed in expert centres, but with the expected spread of the technology to less-experienced centres and operators, availability of such tool may be even more critical.

To address this unmet need, we have developed a finite element (FE) computational simulation algorithm (PlanOp® Structural Heart, PrediSurge, France) to predict the results of TEER interventions. In the present paper, we present our technology and the preliminary results in five patients with secondary MR, highlighting challenges and areas for improvement.

## Methods

### Population

We prospectively enrolled patients with secondary MR referred for TEER in several centres in France and Canada. Inclusion criteria were an atrial or ventricular secondary MR irrespective of comorbidities. Exclusion criteria were insufficient imaging quality precluding segmentation and simulation. As our study was a proof-of-concept and not a feasibility study, only patients with appropriate imaging were analysed and hereby presented. All interventions were clinically indicated, and informed consent was obtained as required by the ethics committees. There was no pre-specified follow-up. The study was approved by each local or national ethics committee. The study was approved by Institutional Review Boards.

### Study design

3D trans-oesophageal echocardiograms (TEEs) performed during the procedure prior to the MV intervention were used to perform the simulation; acquisitions done at the end of the MV intervention served as the reference for comparisons with the results of the simulation. The description of the intervention is outside the scope of the present study but was performed under general anaesthesia and 2D and 3D TEE guidance. All images were obtained under stable haemodynamic conditions.

**Figure 1:**
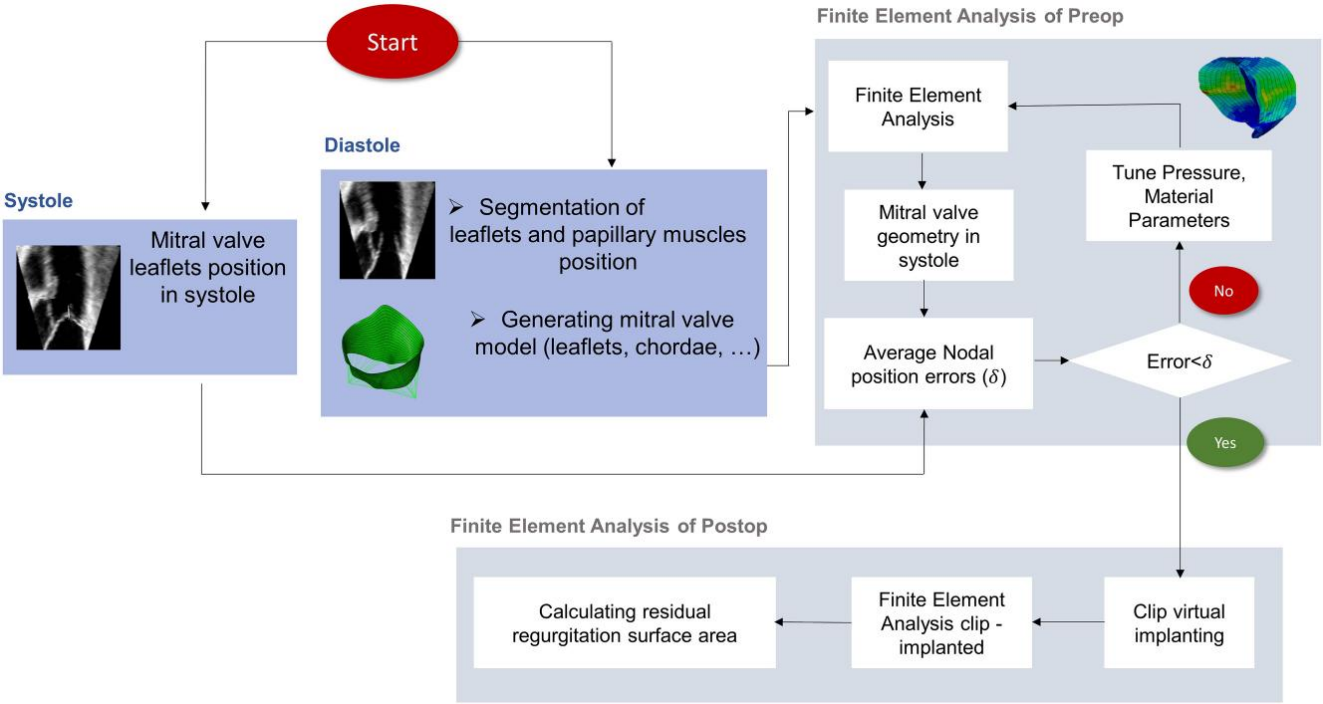
Flowchart depicting the steps of the computational simulated model.

In the first phase, using the en-face 3D-zoom view of the MV including the tips of the papillary muscles, a 3D dynamic model of the MV was obtained. In the second phase, the clip implantation was simulated using the same clip type, size, number, and implantation location that was used during the intervention. The 3D model of the MV obtained after the simulation of the clip implantation was compared with the clinical result obtained at the end of the intervention. We analysed the degree and location of residual MR and the shape and area of the diastolic MV orifice [MV area (MVA)]. Measurements performed using echocardiography and simulated models were performed blinded from each other. The computational simulation steps are presented in *Figure 1*.

### Echocardiographic evaluation

For each patient, MR aetiology and mechanism were assessed. MR severity before and after clip implantation was quantified by a level 3 echocardiographer (first author) using an integrative approach as recommended by guidelines and graded using a 4-grade scale (0 to 4+ MR).^10^ Location and number of MR jets before and after clip implantation were assessed using both 2D and 3D images. The valve area of each orifice was measured using multi-planar reconstruction and summed to obtain the final MVA. Mean pressure gradient (MPG) was measured using continuous wave Doppler.

### Segmentation of the MV before TEER

4D raw ultrasound data sets were acquired using an EPIQ 7 echocardiography system (Philips, Andover, MA, USA) or GE Vivid E95 echocardiography system (GE, Boston, MA, USA). At least one heartbeat was acquired and converted into standard DICOM files to be loaded into an in-house manual segmentation MATLAB code. The valve was first centralized to define a local Cartesian coordinate system by defining the axial axis of the MV. For segmentation of the MV, 3D ultrasound images at end-diastole were divided into 18 cross-sectional slices spaced every 10°. In early systole, we registered two points on the ventricular side of the anterior and posterior annulus. The tips of both papillary muscles were also registered both in end-diastole and early systole. Based on anatomical evaluation of the MV in human cadavers, 12 chordae originating from each papillary muscle, split into 2 branches before inserting on the free margin of the leaflets, were generated.^11^

### FE model

Following the segmentation, data extracted from the TEE images were processed to generate a FE model of the MV. An in-house MATLAB code was developed to create an ABAQUS FE model, and the relevant material properties were assigned to the mitral leaflets and chordae tendineae. The anisotropic hyperplastic Fung strain energy model was employed to model the native MV and during implantation. The function of the Green strain tensor for this model was defined as


$$W=\frac{c}{2}(e^{Q}-1)+\frac{1}{D}\left(\frac{J-1}{2}-\ln J\right),$$

where *W* is the strain energy per unit of reference volume, *c* and *D* are material parameters representing the elasticity and incompressibility level of the corresponding tissue, respectively, *J* is the Jacobian, and *Q* is defined as


Q=ε:b:ε,

where b is a dimensionless symmetric fourth-order tensor of anisotropic material constants that can be temperature dependent, ε is the component of the modified Green strain tensor in which the shear stress is assumed to be negligible, and the leaflets are assumed to be incompressible.^12–14^

### Native valve model

Pressure and chordae pre-stretch were iteratively tuned to enforce the closed leaflet shape as true images of systole. In our native valve validation process, due to the absence of distinct landmarks, we avoided assuming specific material points between open-state and closed-state images or geometries. Rather than relying on point-to-point matching, we leveraged the discernible closed-state geometry of the leaflets, accurately obtained from systolic scans. Subsequently, we developed a method to incorporate and maintain this closed shape throughout FE simulations. When simulating MV closure, iteratively, we penalized any mismatch between the simulated and true closed shapes of the leaflets using a local corrective pressure field developed by Rego *et al*.,^15^ which was at any instance and location linearly proportional to the shortest distance between the FE-based closed valve and the true closed MV surface. A Python code was developed to do this optimization based on the local corrective pressure field, which served to locally ‘push’ regions of the MV. Therefore, the imaged closed-state geometry is matched when the average distance between the FE-based closed valve and the true closed MV surface is below average leaflet thickness. We first defined the spatial domain encompassing the entire vicinity of the true closed MV. Subsequently, we discretized the domain, where we determined the minimum distance between a point on the FE-based closed geometry of the MV and the true MV surface. Based on the computed vector field d (*x, y, z*), the local pressure is corrected as below:


$$p^{n}=p^{n+1}+\delta \alpha \|d\|,$$

where $p^{i}$ is the local pressure in the *i*th iteration of the interested FE and *α* is a correction coefficient that is constant for all the domains. *δ* is 1 or −1 and calculated as


δ=d⋅n/|d⋅n|,

and n is the normal vector of the corresponding element.

### Simulation of the clip implantation

Deformation was applied on the 3D model of the valve to mimic the TEER implantation using the same device type(s), size(s), and position as during the intervention. 3D Slicer version 5.2.1 was used to quantify the residual effective regurgitation orifice (ERO) area of the interventional valve. The systolic simulation geometry was imported into 3D Slicer, and the perimeter of the regurgitation orifice was delineated by selecting various points. In cases of multiple residual jets, areas were summed to calculate the total residual ERO. To determine the MV area of the open orifice, the same method was applied by importing the diastole of the corresponding geometries.

## Results

### Patient characteristics

We prospectively enrolled five patients with secondary MR referred for TEER over approximately a 1-year period. A summary of the clinical and echocardiographic presentations of each patient is provided below and in *Table 1*. Patient 1 was a 75-year-old man with ischaemic cardiomyopathy and reduced left ventricular ejection fraction (LVEF) (35%) with a large scar in the inferior and lateral wall. MR was graded moderate to severe (3+) due to posterior leaflet restriction and pseudo-prolapse of the anterior valve. One MitraClip XTW was implanted, decreasing the MR degree to mild to moderate (2+). Final MVA and MPG were 2.0 cm^2^ and 3 mmHg, respectively. Patient 2 was an 86-year-old man with non-ischaemic (dilated) cardiomyopathy, severe (4+) MR, and posterior leaflet restriction/pseudo-prolapse of the anterior valve. One NTW clip was implanted, decreasing MR to mild (1+); MVA was 2.4 cm^2^, and MPG was 2 mmHg. Patient 3 was a 60-year-old man with non-ischaemic (dilated) cardiomyopathy who initially presented in cardiogenic shock with a severely reduced LVEF (<25%) and 4+ MR. Two XTW clips were implanted with a final MVA of 2.2 cm^2^, MPG of 4 mmHg, and trace MR. Patient 4 was an 85-year-old man with ischaemic cardiomyopathy and prior coronary artery bypass graft in permanent atrial fibrillation. MR was moderate on transthoracic echocardiography but only mild on the TEE at the start of the intervention and was traced after one NTW clip. The final MVA was 2.2 cm^2^ and MPG 1.5 mmHg. Patient 5 was an 81-year-old woman with 4+ atrial functional MR. After one XT clip, MR decreased to 1+ and MVA was 2 cm^2^ and MPG 2 mmHg.

**Table 1:** Patient characteristics before and after MV clip implantation and the comparison of post-implantation echocardiography and simulation results

Patient #	Age, years	Sex	MR grade before clip implantation	Clip number and type	Post-clip implantation				
					Measured MPG, mmHg	Measured MVA, cm^2^	Simulated MVA, cm^2^	Measured MR grade	Simulated ERO, cm^2^
1	75	Male	3+	One XTW	3	2.0	0.70	2+	0.08
2	86	Male	4+	One NTW	2	2.4	2.48	1+	0.08
3	60	Male	4+	Two XTW	4	2.2	1.36	Trace	0.02
4	85	Male	2–3+	One NTW	1.5	2.2	1.62	Trace	0.04
5	81	Women	4+	One XT	2	2.0	1.40	1+	0.02

ERO, effective regurgitant orifice; MPG, mean mitral gradient; MR, mitral regurgitation; MVA, mitral valve area; NTW (W = wide): arm 9 mm and width 6 mm; XT (X = long arm): arm 12 mm and width 4 mm; XTW: arm 12 mm and width 6 mm.

### Results of the simulation

**Figure 2:**
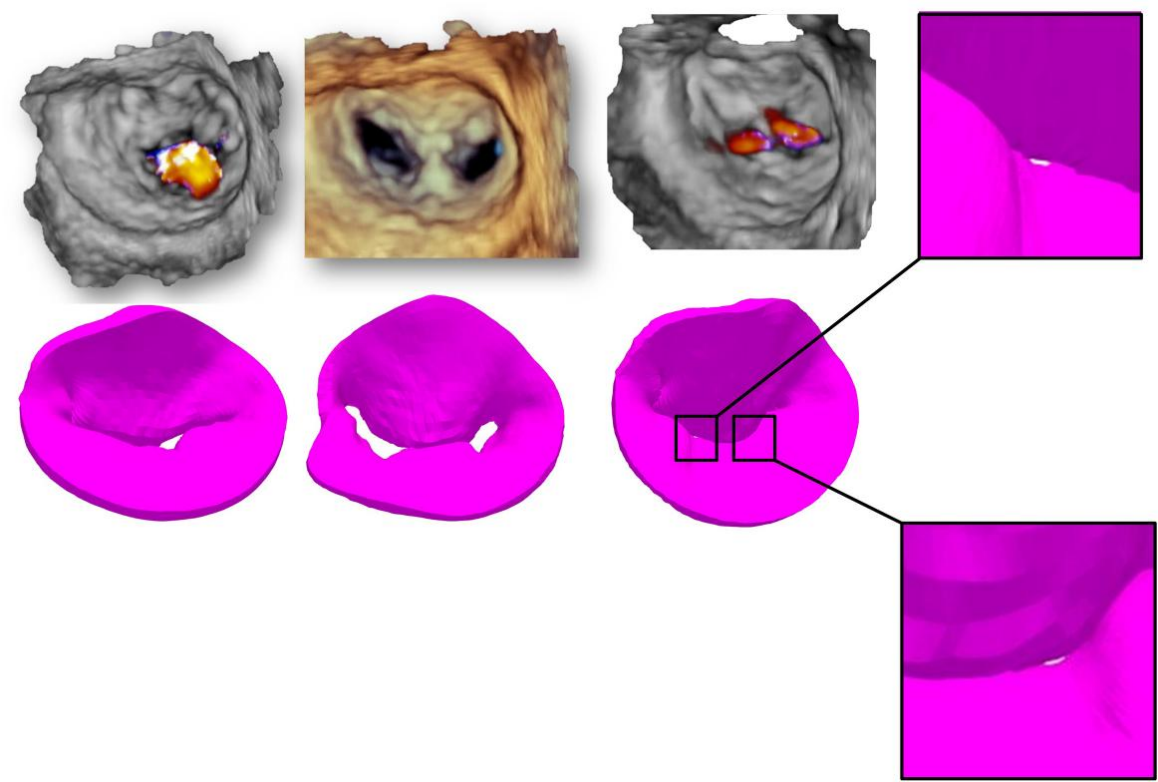
Native 3D TEE images (*top* row) and simulated images (*bottom* row) of the MV in systole before TEER implantation (left column) and after TEER implantation in diastole (*middle* column) and systole (right column) for each of the five patients (Patient 1).

**Figure 3:**
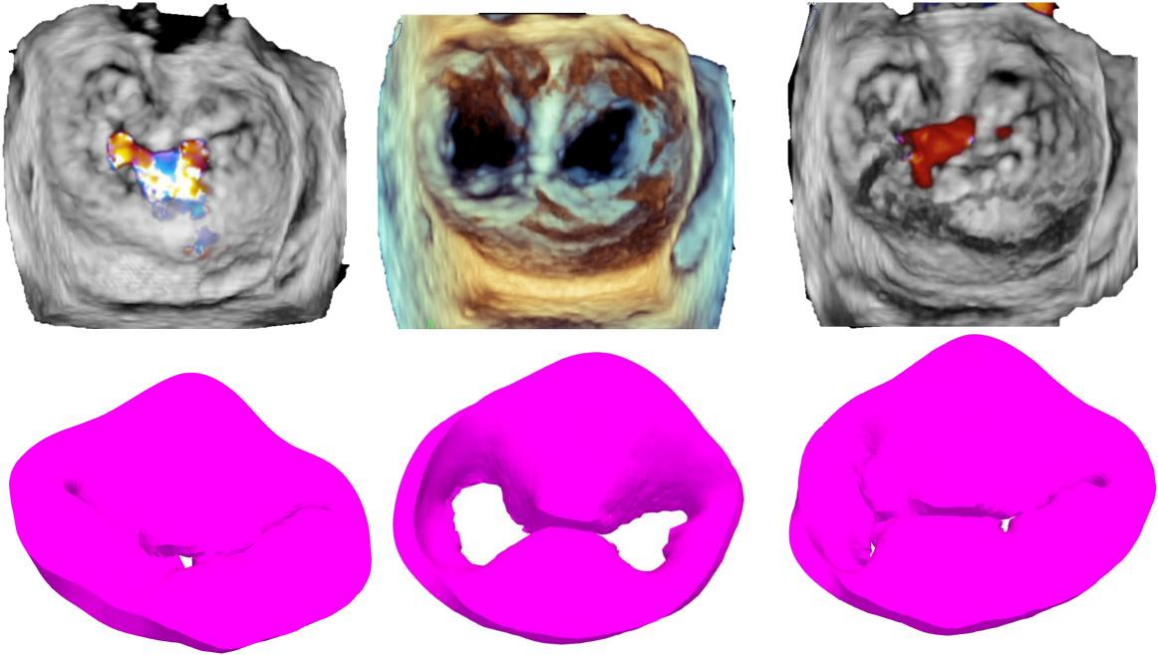
Native 3D TEE images (*top* row) and simulated images (*bottom* row) of the MV in systole before TEER implantation (left column) and after TEER implantation in diastole (*middle* column) and systole (right column) for each of the five patients (Patient 2).

**Figure 4:**
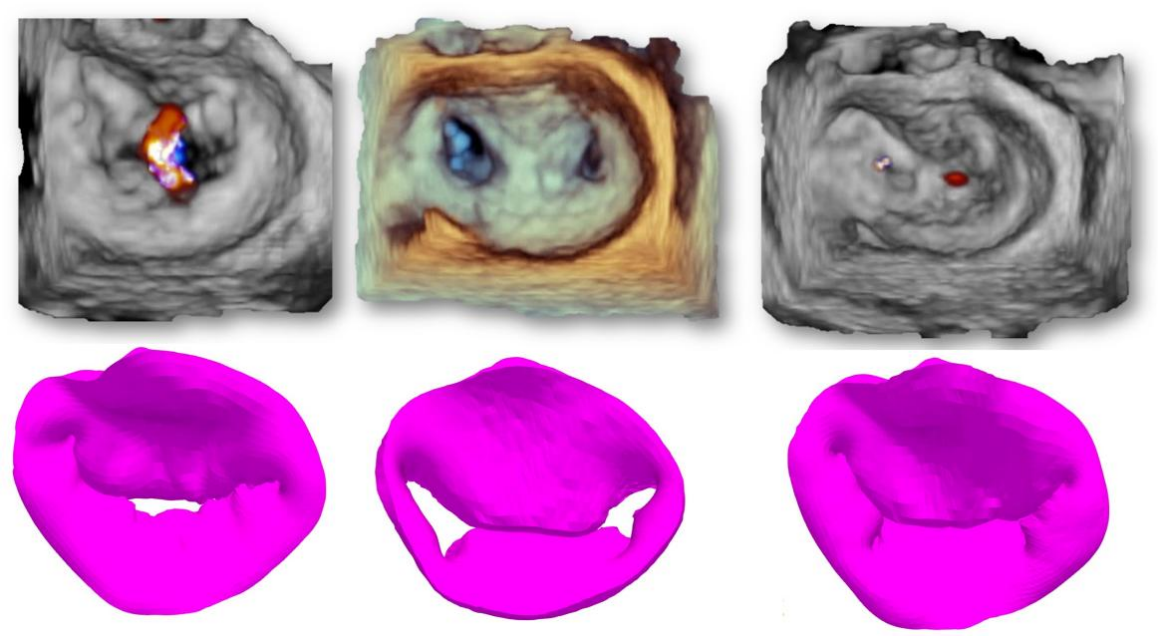
Native 3D TEE images (*top* row) and simulated images (*bottom* row) of the MV in systole before TEER implantation (left column) and after TEER implantation in diastole (*middle* column) and systole (right column) for each of the five patients (Patient 3).

**Figure 5:**
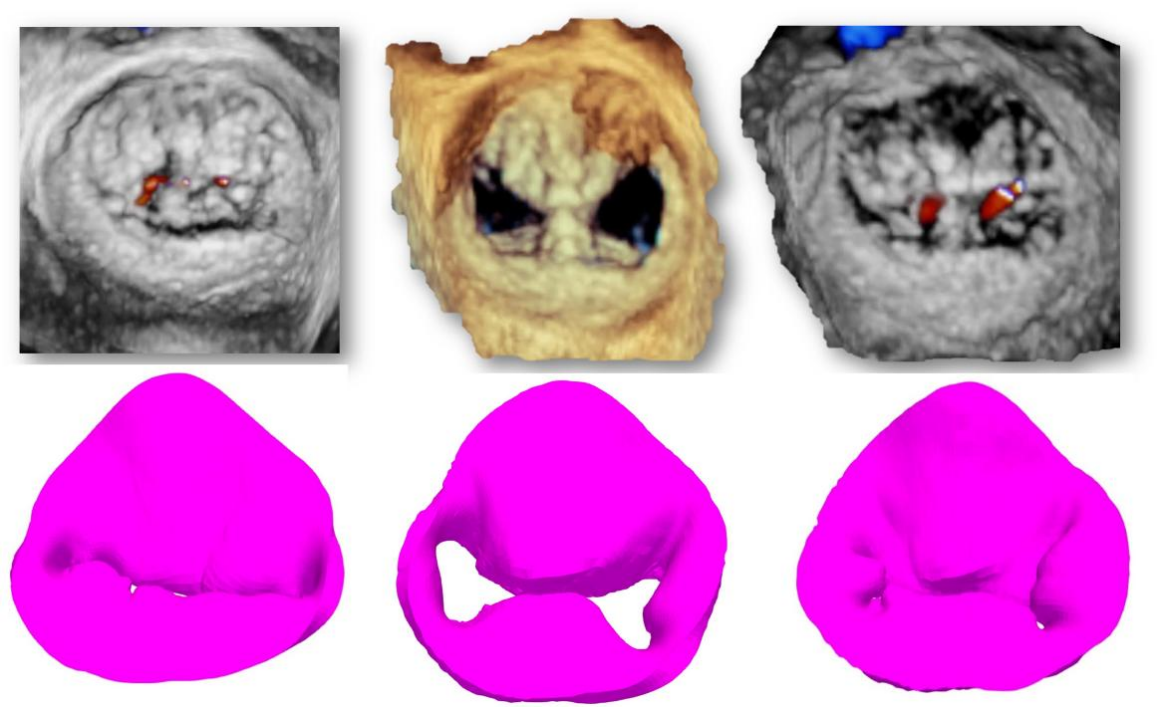
Native 3D TEE images (*top* row) and simulated images (*bottom* row) of the MV in systole before TEER implantation (left column) and after TEER implantation in diastole (*middle* column) and systole (right column) for each of the five patients (Patient 4).

**Figure 6:**
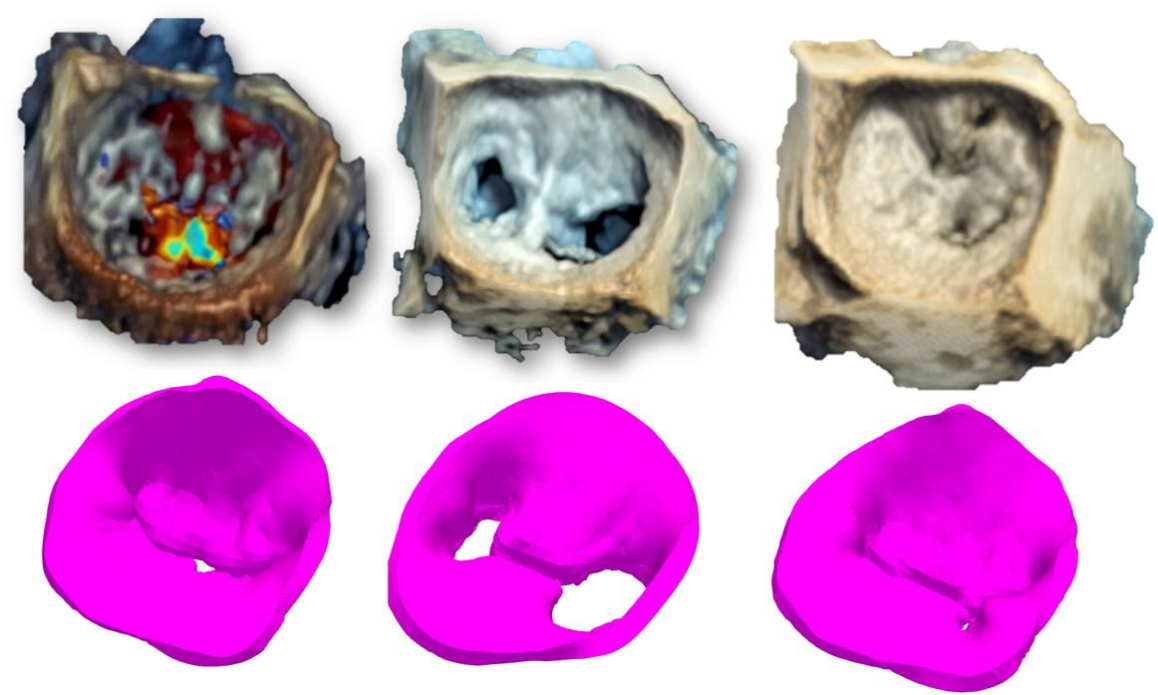
Native 3D TEE images (*top* row) and simulated images (*bottom* row) of the MV in systole before TEER implantation (left column) and after TEER implantation in diastole (*middle* column) and systole (right column) for each of the five patients (Patient 5).

Systolic 3D views of the native valve and the 3D-simulated model for each patient are presented in the left column of *Figures 2–6*. Views of the native valve and of the simulated model after implantation in diastole (middle column) and systole (right column) for each patient are also presented in *Figures 2–6*. As illustrated, the simulation accurately reproduces the shape of the MV orifice after clip implantation. Post-procedural MR degree and MVA obtained from the simulation and from the 3D data set post-implantation are presented in *Table 1*. Overall, there was a good agreement regarding MR degree and location but a trend toward an underestimation of the MVA with the simulation. No statistical analysis was performed due to the small sample size.

## Discussion

The use of artificial intelligence and computational simulation is a rapidly expanding field in cardiovascular intervention, but experience in TEER remains scarce.^9^ In this proof-of-concept study, we present preliminary results regarding our algorithm simulating clip implantation in five patients with functional MR. We show promising results regarding the feasibility and accuracy in terms of predicting residual MR, but the need to improve the estimation of the diastolic MVA.

The initial step of the simulation is to obtain an accurate 3D model of the MV leaflets and apparatus. This step is highly dependent on the quality of the 3D images that are acquired. Segmentation to obtain this 3D model was performed using the en-face 3D loop of the MV and multi-planar reconstruction that currently offers lower temporal and spatial resolution than 2D images. Image quality was overall good for the mid-portion of the MV but was more challenging in the commissural areas even when multi-beat acquisitions were performed. This is an important current limitation but is expected to improve as 3D imaging technology progresses. Fusion images with CT or MRI could be considered as an alternative but are usually not routinely performed before edge-to-edge implantation. These imaging modalities may become more widely available with the emergence of transcatheter valve replacements as an alternative to TEER. It is worth noting that the MV leaflets and annulus were segmented in diastole, while several key reference points as the papillary muscles, annulus, and edge positions of leaflets were registered in systole. The full dynamic 3D model of the MV complex was obtained assuming valve tissue physical properties and applying image-based boundary conditions and pressures on segmented elements to fully track each voxel all along the cardiac cycle.^16,17^ Therefore, for optimization of the native MV, the image-based leaflet geometry in systole was obtained by morphing the open valve using reference points. Such methodology avoids any assumption regarding the tracking of reference points. However, accurately visualizing the edges of the leaflets during systole, especially when leaflets come into contact, poses significant challenges. In addition, tissue properties and pressure were obtained from the literature and therefore not derived from individual patients. We also experienced some bending difficulties near the commissures, which could be improved using patient-specific material properties and pressure in the future.

Once the 3D model was obtained and native valve parameters were validated, we simulated the clip implantation. The simulation was performed based on published clip size and properties. Importantly, we were able to simulate the implantation of one or more clips of various sizes. This is a critical feature enabling operators to select device type, size, and site of implantation offering the optimal predicted result. As technology improves and device armamentarium enriches, they could be added to the toolbox. For all clip implantation, we assumed that 70% of the clip arm’s lengths gripped MV tissue. Further simulations are needed to assess the impact of the length of the grasped tissue on immediate results. Following the simulation of clip implantation, we faced several challenges such as tissue distortion, and we made assumptions regarding annular size reduction. As more patients are collected and more simulations performed, these aspects are expected to improve. The simulation tended to underestimate the final MVA after clip implantation. Tissue thickness strongly influenced MVA calculations and was at least partially responsible for the discrepancy. It is, however, worth noting that echocardiographic measurements of the MVA after clip implantation are often challenging and are also prone to errors. However, the prediction of residual MR is often a bigger concern than the MVA. It is worth noting that currently, we cannot calculate the MPG, but this is definitely a parameter that we aim to obtain in the future. Clinical evaluation of the degree of residual MR was semi-quantitative. The assessment of residual MR post–edge-to-edge intervention is often challenging, and quantitative assessment (ERO) is rarely performed. Our simulation provided direct quantitative measurement of the ERO that compared well with semi-quantitative MR grade using recommended thresholds.^1,2,10^

### Clinical implications and future perspectives

In the present study, the results of mitral TEER simulation in five patients are presented. Our results are far from definitive and should be viewed as a proof-of-concept study with the potential for improvement. Patients were non-consecutive and enrolled based on image quality. Computational simulation needs to be extended to a larger number of patients and to be validated in patients with both primary and secondary MR. In addition, this preliminary work offers important clinical implications beyond the prediction of TEER. This technology could also be applied to transcatheter MV replacement and to transcatheter tricuspid valve interventions. Furthermore, in addition to the prediction of immediate results, our simulated models might provide new indices such as co-aptation height that have been shown to predict MR recurrence and long-term results after surgical MV repair.

## Conclusion

We present proof-of-concept results of computational simulation of mitral TEER in five patients with functional MR and highlight the challenges and limitations of the current technology. Simulation of complex valve interventions for procedural planning and clinical decision-making has been identified as an important unmet need in the field of valvular heart disease. As 3D technology continues to improve and further refinements of our simulation algorithm are achieved, we hope to be able to address this critical gap in care.

## Data Availability

The data underlying this article are intellectual properties of PrediSurge and cannot be shared.
